# Supplementary material for: Patterns of Intron Gain and Loss in Fungi
Source: PLoS Biol. 2004 Nov 30;2(12):e422. doi: 10.1371/journal.pbio.0020422 (PMC532390; doi:10.1371/journal.pbio.0020422)
Supplement: Table S1 — Also available at http://genes.mit.edu/NielsenEtAl/. (4.3 MB ZIP). [file pbio.0020422.st001.zip › NielsenEtAl/html/1398.html]

AN0834.1.NCU00169.1.MG05320.1.FG06644.1


```
 CLUSTAL W (1.82) Multiple Sequence Alignments - Introns Inserted


Sequence 1: NCU00169.1	700 aa
Sequence 2: MG05320.1	704 aa
Sequence 3: FG06644.1	698 aa
Sequence 4: AN0834.1	696 aa
Alignment Length: 711 aa
Number Identitical Residues: 288 aa
Alignment Score (without introns) 16133


MG05320.1 	MSSDYSYDET~GHLWPFFVFTLTTIVTLPLTYALVNRSR-D~PAAKFPRIKTEYKPKHGD
NCU00169.1	MSTDYAYDEE~GHLWPFFVFTLTSIVTVPITYSLIKSSRND~PAAALKRIQTDYKHDQSA
FG06644.1 	MSSDYSYDEQ0GQFFPFFILTLTGIVTLPLTYTLLRPSR-S~DDALAPRIKTDYKPEHAA
AN0834.1  	MSTDYTYDEQ0GQFFPYFVLTLTGLVTFPLTYNLLKPPK-D1LENTAPRIRSDFKPEHED
          	**:**:***  *:::*:*::*** :**.*:** *:. .: .       **::::* .:  

MG05320.1 	LVQSQKSAYKRKN-RTLGLTLFVLGGWAVMGYMLYLISVTEAPVNKLWNPYDILGLSE~T
NCU00169.1	VVDTLRKSEKRKDGSKPWLIALVIAGWAVMGYMIYLIKTTDAPVQHLWNPYDILGIAE~S
FG06644.1 	TVDSLKTARKRSQWRVKRAIFVVIG-WALMAGMVYLIVVTQRTVPKIYNPYDILGISE0S
AN0834.1  	LIEAQKRKRLRKERRIKRIVTVVLG-YAIMAYMAYLIVITARASPKIWDPYDILGVSR0S
          	 ::: :    *.:        .*:. :*:*. * ***  *  .  ::::******::. :

MG05320.1 	ASEKVIKKTYKELSRRLHPDKVKPDPAKNETIESLNDKYVEISKAYQALTDEDVRNNYIQ
NCU00169.1	ATEKQIKKTYKMLSLKFHPDKARPDASKNETIDDLNRRYVEISKAYQALTDEDVRNNFIQ
FG06644.1 	LNEKQIKSHYKRLSLKFHPDKVRPDPAKNETVESLNDFYVELTKAYQSLTDEEVRNNYIQ
AN0834.1  	ANERAITKHYKRLSLLYHPDKIRPDPAKNETIELLNERFVELTKAYKALTDEEVRNNYLQ
          	 .*: *.. ** **   **** :**.:****:: **  :**::***::****:****::*

MG05320.1 	FGHPDGKQGFSINIALPKVIVSDGNGKYVVLVYFMLFGVLLPYWVGSWWYGTQKRSKEGP
NCU00169.1	FGNPDGKQGFSINIALPKAIVSDGNGKYIVLIYFALFGVLLPYLVGSWWYGTLRRSKEGV
FG06644.1 	YGHPDGKQSFSMGIALPQFMVADGSGKYVVLLYTLLLGVLLPWVVGSWWYGTMRMSKEGV
AN0834.1  	YGHPDGKQSFSIGIALPQFIVTEGNGKYVLLVYGGLLGVLLPYIVGKWWYGSQRYTKERV
          	:*:*****.**:.****: :*::*.***::*:*  *:*****: **.****: : :**  

MG05320.1 	LMESANRLFREYEDDIDEGGVISALSSGREFEDVFKGNKADSGLSKIESRILAAGETSQF
NCU00169.1	LMESANRLFKEYQDNIDEGGVVAALSSGQEFEDLFKGDKADAGLSKIESKVMAS------
FG06644.1 	LMESANNLFRQYDDEIEEGGIITALSVGKEFEAVVKGDKIETALSKIESRITADSDCQPT
AN0834.1  	YVASAGNIFREYKDDITDGGIVNALSSGDEFKEAIPAQKAETGLAKLEQKVLADDN----
          	 : **..:*::*.*:* :**:: *** * **:  . .:* ::.*:*:*.:: * ..    

MG05320.1 	AAGMSLKEKEKLDDLDNGVRRKVLDLLWAYLGRIELDDAELTKA~KFEVAPIAHSLTKSF
NCU00169.1	-DELPPKEKKKLEDVENGPRRKALALLWAYLGRIELGDATLEKA~KFQVAPIAHALNKSF
FG06644.1 	ASGMSVKDKDALQDLDNGVRRKALALLWAYLGRVELDDPALTKA~KLQTGPIARALNQSF
AN0834.1  	-KFLTDQEREAIKGMDDLSRRKALALLWAYLGRVELDDPILNGE1KYEVAPIALSLNEAF
          	   :. :::. :..:::  ***.* ********:**.*. *    * :..*** :*.::*

MG05320.1 	TAIALAYGNTAPILASYYASQILIQAIPPKASPLLQLPHFTPSLAKAIEG-DSRVRMSLQ
NCU00169.1	NAIALAYMNTAPLLASYFASQRLIQAIPPKASPLYQLPHFNSAVVRAVEG-DSRVHTTVQ
FG06644.1 	NAITLAYGNISPIANSFYTSQNLVQAVAPHASPLLQLPHITPKIASAIEG-ETKTHMPVH
AN0834.1  	TAVSLAFGNLRPLLGSFRTSQNLIQAVAPGSSPLLQLPHFTDKVVRSIEGEDVKNHFSVQ
          	.*::**: *  *:  *: :** *:**:.* :*** ****:.  :. ::**.: : : .::

MG05320.1 	RFMDQPDSQRRSQAIGDGLLTEAQYKEAVEVAKQLPFLRVAKAYFKVTGEKYIIPSSLVT
NCU00169.1	AFMDQPDAKRRSLVVGKGLLTDAQYKETIAYAKQLPYLRVAKAFFKVTGEKFIIPSSLVT
FG06644.1 	RFMDRPDAQRRQMAIGEGLLTEEQYETAIGVAKQIPFFRVCRAFFKVTGERFITPSSLVS
AN0834.1  	KFMDMPEEKRRSLTVGAGLLSEQQYTTAITVAKQLPVLKISKAWFKVMGEKVITPSSLVQ
          	 *** *: :**. .:* ***:: **  ::  ***:* :::.:*:*** **: * ***** 

MG05320.1 	LVVKGRFVPPGSENVPEIVGTDLEDVDPAEDDLDAILGRK--KKQVGKDERGKPVYGADD
NCU00169.1	LVVKGRFVPPGSEKVPDVTPIDLEDIDPAEDDLDAILGRKKTKKVVGKDEKGKPIYEESA
FG06644.1 	LVIKGRFIPPGTDDVPAINELDLEDIDPAEDDIEAINGRKK-KTIKGPDGKLIPIEEES-
AN0834.1  	LVVKARFIPPGSKDVPEPDPADLEDIDPEEGDLDALMGRGP-----SRNRSVKFADGQNN
          	**:*.**:***:..**     ****:** *.*::*: **       . :         . 

MG05320.1 	ETP-VVPPLAYAPYFARDHSPRWNVFLTDSKQGKMAVPPFTFAQFDKPIFKDDGKTPTYE
NCU00169.1	ESEDVYPPLASSPYFARDHSPRWHVFLTDSKQGRVAVPPFTFVSFDRPIYESDGKTPTFA
FG06644.1 	----ILPPLTHAPYYARDHSPQWHAFLSDSKQGKMAVPPFHFAKFDQPIIDDEGK-PTFN
AN0834.1  	VNKKVQPPLAHAPYLALDHSPRWQIFLVDGKQGKIAVPPFTFTTFEKPMLDEAGN-PTYN
          	 . .: ***: :** * ****:*: ** *.***::***** *. *::*: .. *: **: 

MG05320.1 	MQTLKAQFQAPPQAGHYTFVMHVVCDSYVGFDTKMEVTLVVDEASRAAEMTDEEEISEPD
NCU00169.1	MQTMKAQFQAPPQAGHYTFVMHVVCDSYVGFDTKMEVTLVVEDASRAAEMKDEEEISEPE
FG06644.1 	MQTLKAQFAAPPQAGHYTFVLHVICDAYVGFDTKMEVTLVVEEASKAAEMVQEDEISEPE
AN0834.1  	VQTLRMQFQAPPQVGDFHFVMHMVCDSYIGLDSKLDITLHIDDPAKAAALEEEDDISEPD
          	:**:: ** ****.*.: **:*::**:*:*:*:*:::** :::.::** : :*::****:

MG05320.1 	ED1SIAGIMSAAKGGAPPPRKKKQAVEEDESDDESGT-EEEADDTSDTNTDTEDES
NCU00169.1	ED1SIAGIMNAAKG---APTKQRVVKDEEDSDEESGTDDDEDDDTSDTNTDTEPEN
FG06644.1 	ED1SLAGQMHVLKTGQTPNTRRK---DSDDSDDESGTDEEEDDDTSATNTDTEDES
AN0834.1  	ED1SIAGQMQALKTGQ-PPKKKAKKPADDSSDEESDTEGDEGD-TSDTNTETDVDD
          	** *:** * . * .  .  ::     .:.**:**.*. :* * ** ***:*: :.
```
